# Elucidating shared genetic signals between type 2 diabetes and three neurodegenerative dementia phenotypes

**DOI:** 10.1016/j.xhgg.2026.100667

**Published:** 2026-09-01

**Authors:** Ana Luiza Arruda, Archit Singh, Ozvan Bocher, Benjamin F. Voight, Andrew P. Morris, Eleftheria Zeggini

**Affiliations:** 1 Institute of Translational Genomics, Helmholtz Zentrum München – German Research Center for Environmental Health, 85764 Neuherberg, Germany; 2 Medical Research Council (MRC) Epidemiology Unit, University of Cambridge, Cambridge, UK; 3 Munich School for Data Science (MUDS), Helmholtz Zentrum München – German Research Center for Environmental Health, 85764 Neuherberg, Germany; 4 Doctoral Program of Experimental Medicine and Health Sciences, TUM School of Medicine and Health, Technical University of Munich, 81675 Munich, Germany; 5 Centre for Genomic and Experimental Medicine, Institute of Genetics and Cancer, University of Edinburgh, Edinburgh EH4 2XU, UK; 6 University Brest, Inserm, EFS, UMR 1078 Brest, France; 7 Corporal Michael J Crescenz VA Medical Center, Philadelphia, PA, USA; 8 Department of Genetics, University of Pennsylvania Perelman School of Medicine, Philadelphia, PA, USA; 9 Institute for Translational Medicine and Therapeutics, University of Pennsylvania Perelman School of Medicine, Philadelphia, PA, USA; 10 Department of Systems Pharmacology and Translational Therapeutics, University of Pennsylvania Perelman School of Medicine, Philadelphia, PA, USA; 11 Centre for Genetics and Genomics Versus Arthritis, Centre for Musculoskeletal Research, Division of Musculoskeletal and Dermatological Sciences, The University of Manchester, Manchester, UK; 12 TUM University Hospital, TUM School of Medicine and Health, Technical University of Munich (TUM), 81675 Munich, Germany

**Keywords:** comorbidity, shared genetic etiology, type 2 diabetes, Alzheimer disease, Lewy body dementia, frontotemporal dementia, multi-omics, lipid metabolism

## Abstract

Type 2 diabetes (T2D) and dementia frequently co-occur, yet the biological mechanisms underlying this comorbidity remain incompletely understood. Here, we systematically investigate shared genetic signals between T2D and three forms of neurodegenerative dementia (Alzheimer disease, Lewy body dementia, and sporadic frontotemporal dementia) using large-scale genome-wide association studies of clinically diagnosed individuals. We identify five genomic regions harboring shared association signals between T2D and at least one dementia subtype. Among these, the *APOE* locus was common to all dementia subtypes, whereas the remaining four loci (*GBA*, *CRY2*/*PEX16*/*MAPK8IP1*, *INO80E*, and *NSF*) were each shared exclusively between T2D and one dementia subtype. Integrating multi-omics data across several disease-relevant tissues and orthogonal lines of functional evidence, we prioritize 26 candidate genes through which these shared genetic loci potentially mediate their effect. Pathway enrichment highlights lipid and lipoprotein regulatory biology as a central shared axis. Mendelian randomization analyses using genetically regulated gene expression in relevant tissues indicate pleiotropic mechanisms with divergent phenotypic consequences. Our findings identify shared genetic loci between T2D and neurodegenerative dementia, revealing systemic metabolic-neurodegenerative trade-offs and highlighting key genes that underpin the comorbidity, providing a framework for improved understanding of age-related multi-morbidity.

## Teaser

We identify limited, subtype-specific genetic overlap between type 2 diabetes and neurodegenerative dementias and prioritize effector genes. Some of the shared genetic loci exhibit opposing effects on metabolic and neurodegenerative disease risk, suggesting that localized pleiotropy and biological trade-offs, rather than broadly shared genetic susceptibility, underlie this age-related comorbidity.

## Introduction

As life expectancy continues to increase worldwide, the number of people who develop multiple age-related conditions is growing, amplifying their combined impact on individual well-being and healthcare systems.^1^ Dementia is a prevalent prototypical age-related neurodegenerative condition defined by progressive decline in cognitive function severe enough to interfere with daily living and independence. Dementia affects over 55 million people worldwide and is projected to increase in prevalence in the coming decades.^2^ In 2019, the global economic cost of dementia was estimated to be over US$1 trillion.^3^

The most common form of dementia, accounting for 80% of cases, is Alzheimer disease (AD [MIM: 104300]).^4^^,^^5^ While increasing age is the primary risk factor for AD, genetic and environmental factors also play a role, most notably the apolipoprotein E (*APOE* [MIM: 107741]) ε4 allele and cardiovascular health.^6^ Twin-based studies indicate a substantial heritability component, with genetic factors estimated to contribute approximately 60%–80% of the overall disease risk.^7^ Lewy body dementia (LBD [MIM: 127750]) accounts for 3%–7% of dementia cases and is characterized by the accumulation of α-synuclein aggregates.^8^ Sporadic frontotemporal dementia (FTD [MIM: 600274]) is comparatively rare, accounting for less than 3% of all dementia cases, although it constitutes a higher proportion of early-onset dementia.^9^ FTD comprises a heterogeneous group of disorders characterized by progressive degeneration of the frontal and temporal lobes in the brain and is primarily associated with tau, TDP-43, or FUS proteinopathies. The most common form is sporadic FTD, accounting for approximately 70% of FTD cases and arising in the absence of a known familial mutation.^10^ In contrast to the neurodegenerative dementia subtypes introduced, vascular dementia primarily originates from peripheral and cerebrovascular systems rather than intrinsic neurodegenerative processes of the central nervous system. It is characterized by cognitive impairment resulting from macro- and microvascular damage due to reduced cerebral blood flow, often caused by vessel constriction or occlusion.^4^

Several population-based studies have reported an association between dementia and age-related cardiometabolic conditions, including diabetes. Type 2 diabetes (T2D [MIM: 125853]) is the most common form of diabetes and is characterized by hyperglycemia, insulin resistance, and impaired glucose regulation.^11^ In 2021, T2D had a global prevalence of over 536 million individuals affected with an increasing tendency, attributed partly to the rise in sedentary lifestyles and obesity.^12^ Similarly to dementia, T2D is a complex disease influenced by a combination of genetic and environmental factors, with heritability estimated at 50%.^13^

Multiple observational studies have shown evidence linking T2D and various forms of dementia^14^^,^^15^^,^^16^^,^^17^ and cognitive decline.^16^^,^^18^ A recent meta-analysis of over 9 million individuals reported a 1.43-fold increased risk for all-cause dementia among individuals with diabetes.^19^ Mechanistically, dysfunctional glucose metabolism and insulin signaling have been implicated in AD,^20^^,^^21^^,^^22^ leading some researchers to describe it as “type 3 diabetes.”^23^ Beyond insulin dysregulation, additional biological processes contribute to neurodegenerative dementia and T2D comorbidity. Clinical imaging and neuropathology studies show that T2D is associated with small-vessel disease, white-matter lesions, and cerebral infarcts that increase dementia risk.^24^ Preclinical experimental work in cellular and animal models demonstrates that hyperglycemia, atherogenic lipid profiles, and disrupted cholesterol homeostasis drive oxidative stress, mitochondrial dysfunction, and neuroinflammation, promoting amyloid accumulation, tau pathology, and synaptic loss.^25^^,^^26^^,^^27^ Collectively, these processes disrupt neuronal homeostasis and accelerate neurodegeneration.

Genetic studies have pointed to a partial shared genetic architecture underlying this comorbidity pair.^28^^,^^29^ Previous studies have found a risk-increasing association between a T2D genetic score and vascular dementia, but not for AD.^30^^,^^31^^,^^32^ Metabolic mechanisms were found to be important for the comorbidity between vascular dementia and T2D.^33^ Leveraging data from large-scale genome-wide association studies (GWASs), we sought to elucidate the shared genetic architecture underlying the comorbidity between T2D and neurodegenerative dementia. By integrating genetic association, functional genomics, and multi-omics data across disease-relevant tissues, we identify shared loci and prioritize candidate effector genes with potential therapeutic relevance. Our findings reveal limited subtype-specific shared genetic architecture, providing insights into the biological relationship between metabolic disease and neurodegeneration.

## Material and methods

### Ethics

This study used only publicly available summary statistics from previously published GWASs and molecular quantitative trait locus (QTL) datasets. No new human participants were recruited, and no individual-level data were accessed. All contributing studies obtained approval from their respective institutional ethics committees and informed consent from participants, as described in the original publications.

### GWAS summary statistics

We investigated the shared genetic etiology of neurodegenerative dementia and T2D, using publicly available GWAS data. For AD, we used the largest European ancestry GWAS meta-analysis with clinical and autopsy-documented cases, i.e., without proxy cases, comprising data from 21,982 affected individuals and 41,944 control subjects.^34^ For LBD, we used a European ancestry GWAS meta-analysis of 2,591 diagnosed individuals and 4,027 neurologically healthy control individuals.^35^ For sporadic FTD, the European ancestry GWAS meta-analysis that we used consisted of 3,756 clinically diagnosed individuals and 11,233 control subjects.^36^ We set the threshold for genome-wide significance at *p* value of <5 × 10^−8^ for all disease GWASs. For T2D, we used the latest published European ancestry GWAS meta-analysis from the Type 2 Diabetes Global Genetics Initiative (T2DGGI) that encompasses 242,283 affected individuals and 1,569,734 control subjects.^37^

We also used GWAS summary data from adiposity-related traits from the Global Lipids Genetics Consortium (GLGC).^38^ The GWAS sample size for contributing cohorts similar to reference populations obtained in Europe was 1,320,016, and we used data from the following five blood lipid traits: low-density lipoprotein cholesterol (LDL-C), high-density lipoprotein cholesterol (HDL-C), triglycerides (TGs), total cholesterol (TC), and non-HDL-C.

### Molecular QTL summary statistics

We defined a set of tissues biologically relevant to dementia and T2D used for downstream analyses. All available brain tissues were used for all forms of dementia investigated here. For T2D, we selected pancreatic islets, liver, subcutaneous and visceral adipose tissues, skeletal muscle, and the brain cortex. We then obtained datasets from bulk and single-cell analyses from various molecular QTL mapping studies in these tissues, including expression (eQTL), protein (pQTL), and splicing (sQTL). A list of the molecular QTL datasets used, along with sample sizes, can be found in Table S1. Genomic mapping from GRCh38 to GRCh37 was performed using the CrossMap R package (v.0.5.4) for all eQTL datasets from MetaBrain and the fetal brain eQTL dataset. To ensure compatibility, we conducted genomic mapping from GRCh38 to GRCh37 using the liftOver function from the rtracklayer R package (v.1.22.0).

### Genetic correlation analysis

First, we estimated genome-wide genetic correlations between T2D and the investigated dementia subtypes via linkage disequilibrium score regression (LDSC)^39^ (Table S2). GWAS summary statistics were harmonized using munge_sumstats.py. Analyses were restricted to HapMap3 variants and performed using European 1000 Genomes Phase 3 linkage disequilibrium (LD) scores.^40^

Second, polygenic overlap between T2D and the investigated dementia subtypes was estimated using MiXeR v.2.2.1^41^ (Table S3). Summary statistics were reformatted into MiXeR-compatible files and analyzed using the European 1000 Genomes Phase 3^40^ LD reference panel distributed with MiXeR. For each T2D-dementia pair, univariate models were first fitted for each trait, followed by bivariate MiXeR models to estimate trait-specific and shared polygenicity. Analyses were repeated across 20 independent random subsets of approximately 2 million LD-pruned variants, and replicate estimates were combined. Model support was evaluated against constrained minimum- and maximum-overlap models using the Akaike information criterion (AIC) and Bayesian information criterion (BIC).

### Bidirectional causal inference between diseases using Mendelian randomization

Bidirectional two-sample Mendelian randomization (MR) analyses were performed to investigate potential causal relationships between T2D and each dementia subtype (AD, FTD, and LBD) (Table S4). Instrumental variables (IVs) were defined as LD-based clumped genome-wide significant (*p* <5 × 10^−8^) variants with *r*^2^ < 0.001 over a 10-Mb window. The IVs were then harmonized between exposure and outcome datasets using the *TwoSampleMR* R package.^42^ The random effects inverse-variance weighted (IVW) method was used as the primary analysis. Sensitivity analyses included MR-Egger regression, weighted median, MR-PRESSO, and Steiger filtering to assess the robustness of the findings and potential violations of MR assumptions, including horizontal pleiotropy and reverse causation.^43^^,^^44^ Cochran’s Q statistic was used to assess heterogeneity, and the MR-Egger intercept was used to test for directional pleiotropy. *p* values were adjusted for multiple testing using the Benjamini-Hochberg false discovery rate (FDR).

### Genetic colocalization analysis

We defined 1-Mb genomic regions centered on independent genome-wide significant independent signals identified in the GWAS summary statistics for each dementia form and for T2D. We have not considered signals in the HLA region (chr6:28477797–33448354, GRCh37), due to its complex genomic structure characterized by strong, long-range LD. For each dementia form, we then performed regional, pairwise genetic colocalization analyses with T2D using the estimated regression coefficients (effect sizes) and standard errors from the respective GWAS (Table S5). The first set of analyses were conducted using the *coloc*.*abf* function from the *coloc* R package (v.3.2.1).^45^ This function calculates the posterior probabilities for five mutually exclusive association hypotheses under the assumption that each trait has a single causal variant within the region. The hypotheses tested in a genetic colocalization framework are summarized as follows.

H0: no trait has a genetic association in the region
H1: trait 1 has a genetic association in the region
H2: trait 2 has a genetic association in the region
H3: both traits have a genetic association in the region, but with different causal variants
H4: both traits share a genetic association (single causal variant) in the region

The single-variant assumption of *coloc*.*abf* is not always appropriate for polygenic complex traits. Thus, we conducted additional pairwise conditional and colocalization (PWCoCo) analyses in regions that did not show evidence of colocalization using the conventional *coloc*.*abf* approach.^46^ In brief, this method performs conditional analysis using GCTA-COJO to identify independent signals for each trait in the analyzed genomic region and subsequently performs colocalization using *coloc*.*abf* for each pair of conditionally independent signals. We used the 1000 Genomes Project European population as the LD reference panel for the conditional analysis.^40^ We considered a signal as colocalized if the adjusted data was genome-wide significant for at least one trait.

We used the default prior probabilities of the *coloc* R package for our analyses and considered evidence for colocalization if the posterior probability of a shared causal variant associated with H4 (PP4) >0.8. For the *coloc*.*abf* results, in addition to the posterior probability, we calculated a 95% credible set for the causal variant by taking the cumulative sum of the variants’ posterior probabilities to be causal, conditional on H4 being true. This could not be calculated for the PWCoCo results, as the software does not provide a PP4 per tested variant. LD between the estimated lead causal variant and the other variants in the colocalized region was calculated using PLINK (v.2.0 alpha)^47^ based on the European ancestry UK Biobank data^48^ and was used for visualizing the results in regional association plots.

### Multi-trait genetic colocalization analysis with molecular QTL data

To gain insight into the biological mechanisms underlying the shared genetic signals between dementia and T2D identified through genetic colocalization analysis, we conducted multi-trait genetic colocalization analyses incorporating molecular QTL data (Table S6). This approach enabled the identification of genetic variants that influence molecular traits and may represent links to specific dementia forms or T2D. The analyses integrated three sets of summary statistics: dementia GWAS, T2D GWAS, and molecular QTL data derived from disease-relevant tissues or cell types (Table S1). All analyses were performed within a 1-Mb window centered on the lead variant identified in the initial dementia-T2D colocalization analysis.

For this analysis, we applied a multi-trait genetic colocalization framework implemented in the R package *HyPrColoc* (v.0.0.2).^49^ Similar to the *coloc*.*abf* function in the *coloc* package, *HyPrColoc* assumes the presence of a single shared causal variant per locus and estimates posterior probabilities for five colocalization hypotheses (as described in the previous section). To evaluate colocalization across T2D, a subtype of dementia, and a molecular QTL, we disabled the Bayesian divisive clustering algorithm (*bb*.*alg* = FALSE). Based on prior evidence indicating colocalization between dementia and T2D within the genomic regions under investigation, we adjusted the prior probabilities accordingly. Specifically, the parameter *prior*.*1*, which denotes the prior probability that a variant is associated with a single trait, was kept at its default value of 1 × 10^−4^. The parameter *prior*.*c*, representing the conditional probability that a variant is associated with an additional trait given association with one trait, was increased from the default value of 0.02–0.05 to reflect this prior knowledge. We defined evidence of colocalization as a PP4 >0.8. To further strengthen the inference, we required that the 95% credible set derived from the *HyPrColoc* analysis overlap by at least one variant with the 95% credible set from the corresponding dementia-T2D colocalization analysis.

To evaluate the robustness of the HyPrColoc results to the prior specification, we repeated the colocalization analysis for all colocalized signals (PP4 > 0.8) under prior.c = 0.05 using prior.c values of 0.01, 0.02, and 0.10 while keeping prior.1 fixed at 1 × 10^−4^. Posterior probabilities were compared across priors. As expected, decreasing prior.c reduced the magnitude of the posterior probabilities. All 22 colocalized signals identified in the primary analysis (prior.c = 0.05) remained at prior.c = 0.10. Using the default value (prior.c = 0.02), only one signal remained above the predefined threshold (PP > 0.8), while the remaining 21 retained posterior probabilities above 0.7 (Table S7). No additional colocalization signals were identified under the alternative prior specifications. These findings indicate that the choice of prior.c primarily influences the magnitude of the posterior probability rather than identifying different colocalization signals.

### Differential gene and protein expression

For genes located near genomic regions that colocalized between dementia and T2D GWASs, we performed a systematic lookup using publicly available summary statistics from transcriptomic and proteomic studies related to dementia and T2D. The objective was to determine whether genes proximal to shared genetic signals also showed significant differential expression in independent datasets, thereby providing orthogonal supportive evidence of disease relevance. Differential expression was not interpreted as evidence of causal involvement in disease pathogenesis. Because the transcriptomic and proteomic datasets were generated by independent studies with different sample sizes, tissues, experimental designs, and analytical pipelines, we retained the significance thresholds and fold-change criteria defined by the original studies wherever possible. Differential expression was used as an independent line of supporting evidence during gene prioritization, and no direct comparisons of effect sizes or significance across datasets were performed.

For AD, we retrieved RNA-sequencing-based differential expression results from postmortem superior temporal gyrus tissue, comprising 24 individuals with AD and 38 cognitively normal control subjects.^50^ Differential expression was assessed using linear regression, adjusting for sex, diagnosis, and surrogate variables. Genes were defined as differentially expressed if they showed a fold change >1.2 in either direction and an FDR <0.05. We further analyzed transcriptomic data from temporal (268 AD individuals and 203 control subjects) and frontal lobes (417 AD individuals and 335 control subjects).^51^ Covariates included postmortem interval, RNA integrity number (RIN), age at death, and sex. In the original analysis, genes were considered differentially expressed if they had an FDR <0.01 and a fold change exceeding 1.3 in either direction. To extend the analysis to other dementia forms, we incorporated gene-expression data from the anterior cingulate cortex of 436 individuals with LBD, 53 individuals with AD, and 81 healthy control individuals.^52^ The regression model included sex, batch, proportions of reads aligning to coding, intronic, and intergenic regions, RIN, and ENO2 expression as a neuronal biomarker to account for disease-related neuronal loss. Genes were classified as differentially expressed if their absolute log_2_ fold change exceeded 0.25 and with FDR <0.05. Finally, we integrated plasma proteomics data from individuals with AD and FTD, comprising 1,966 AD individuals, 175 FTD individuals, and 5,879 control subjects.^53^ Differential protein abundance was assessed separately in each cohort using linear regression, with protein abundance as the dependent variable and disease status as the independent variable, adjusting for age and sex. Cohort-specific estimates were combined using fixed-effects meta-analysis. In our analysis, proteins were considered differentially abundant if they met an FDR threshold of <0.05.

For T2D, we analyzed gene-expression data from pancreatic islets obtained from 57 metabolically phenotyped pancreatectomized individuals, including 39 individuals with T2D and 18 non-diabetic control subjects.^54^ Differential expression was assessed using a linear regression model adjusted for age, sex, and body mass index (BMI). In the original study, genes were considered differentially expressed if they exhibited a fold change greater than 1.5 in either direction and an adjusted *p* value of <0.05. Additionally, we used gene-expression data from liver tissue collected from 18 individuals, including 9 with T2D.^55^ Differential expression was originally defined using a fold-change threshold of >2 and a nominal *p* value of <0.05. For consistency across analyses, we applied FDR correction and classified genes as differentially expressed if they showed a >2-fold change in either direction and an adjusted *p* value of <0.05.

### Rare and syndromic human diseases

For genes located near genomic regions that colocalized between dementia and T2D GWASs, we conducted a systematic query of the Online Mendelian Inheritance in Man (OMIM) database (https://omim.org/) using its application programming interface (API). The objective of this analysis was to identify rare and syndromic human diseases associated with these genes that exhibit clinical features relevant to dementia or T2D. Diseases were considered dementia related if they exhibited phenotypes involving behavioral or psychiatric abnormalities and/or alterations in brain morphology. Likewise, diseases were classified as T2D related if they included phenotypes associated with hyperglycemia or metabolic dysfunction. The complete lists of rare and syndromic human diseases associated with T2D- and dementia-related phenotypes are provided in Table S8.

### Knockout mouse phenotypes

For genes located near genomic regions that colocalized between the dementia and T2D GWASs, we conducted a systematic interrogation of knockout mouse databases. The objective of this analysis was to identify genes whose disruption results in phenotypes relevant to either dementia or T2D, thereby providing functional evidence for their potential involvement in disease mechanisms.

We first curated comprehensive lists of phenotype terms associated with dementia and T2D. For T2D, these included phenotypes related to insulin regulation and glucose metabolism, encompassing terms such as insulin, glucose, diabetes, hyperglycemia, pancreas, pancreatic, obesity, BMI, body weight, body mass, body fat, beta cell, and glucosuria. For dementia, phenotype terms were selected to capture alterations in brain and craniofacial morphology, behavioral and psychiatric traits, and neuronal structure and function. The complete lists of dementia- and T2D-related phenotypic terms are provided in Table S9.

The analyzed databases include the International Mouse Phenotyping Consortium (IMPC) (https://www.mousephenotype.org/), Mouse Genome Informatics (MGI) (http://www.informatics.jax.org/), and Rat Genome Database (RGD) (https://rgd.mcw.edu/). For IMPC and MGI, knockout phenotypes were retrieved using batch query tools to systematically extract phenotype annotations for each gene. For RGD, phenotype information was obtained through programmatic access using the available API.

### Gene prioritization framework

First, we extracted all genes located within ±1 Mb (2 Mb total) genomic regions that colocalized between dementia and T2D. Each gene was then evaluated using multiple, complementary biological lines of evidence derived from multi-omics and functional genetics data.

Specifically, we assessed four primary lines of evidence.

(1) Multi-trait genetic colocalization with molecular QTLs from disease-relevant tissues
(2) Differential gene expression or protein abundance in disease-relevant tissues comparing affected individuals with healthy control individuals
(3) Functional evidence from knockout mouse models exhibiting phenotypes related to dementia or T2D
(4) Evidence from rare or syndromic human diseases with phenotypes relevant to dementia or T2D

Using these criteria, we constructed two independent scores for each disease: one reflecting evidence for dementia involvement and one for T2D involvement.

As an additional independent line of evidence, we examined whether the lead colocalization variants and, where available, all variants within the 95% credible sets of colocalized regions were missense variants affecting any of the analyzed genes, based on Ensembl annotations.^56^ The total gene score was defined as the sum of the dementia score, the T2D score, and the missense variant score. However, if a missense variant constituted the only source of evidence for a gene, the total score was retained at zero, as this information alone does not directly support involvement in the shared comorbidity.

In total, five orthogonal biological lines of evidence were integrated to score each gene. Genes receiving at least one point for both the dementia and T2D scores were classified as potential effector genes implicated in the comorbidity between the two diseases. Within this group, genes with a total score of ≥3 were prioritized.

### Pathway analysis

We performed an over-representation analysis on the set of the genes prioritized by our genomics data-driven approach to be implicated in the comorbidity between dementia and T2D. Pathway enrichment was carried out using the ConsensusPathDB enrichment framework (http://cpdb.molgen.mpg.de/), which integrates curated information from multiple pathways including Gene Ontology.^57^ Gene Ontology terms were considered across molecular function, biological process, and cellular component categories, up to level 5. A minimum overlap of two genes was applied in the enrichment analysis, and the significance threshold was set at FDR ≤ 0.05.

### Investigation using circulating lipid GWAS meta-analyses

We next assessed whether the lead colocalization variants and, where available, all variants within the 95% credible sets of regions shared between dementia and T2D were genome-wide significantly associated (*p* <5 × 10^−8^) with lipid-related traits. The lipid traits examined included LDL-C, HDL-C, TGs, TC, and non-HDL-C. Specifically, we utilized summary statistics from the European-ancestry subset of the GWAS meta-analysis conducted by the GLGC.^51^ For shared colocalized regions in which none of the variants reached genome-wide significance for lipid traits, we further investigated whether these variants were in high LD with genome-wide significant index variants associated with lipid traits. LD was assessed using the R package *LDlinkR* (v.1.4.0), which interfaces with the web-based LDlink tool and uses the 1000 Genomes European population as the reference panel.^40^^,^^58^

### Causal inference analysis between molecular QTL and GWASs

For genes where corresponding genomic loci show evidence of colocalization between molecular QTL GWAS, neurodegenerative dementia GWAS, and T2D GWAS, we next assessed whether the QTL regulating gene expression has a causal effect on disease risk using two-sample MR analyses.^59^ To this end, we performed MR analyses using the *TwoSampleMR* R package (v.0.6.22) curated by MR-Base,^42^ treating QTL summary statistics from disease-relevant tissues as exposures and disease GWAS summary statistics as outcomes.^42^ When multiple QTL-gene pairs were available for a given gene, we applied LD-based clumping to select independent genetic instruments. For QTL datasets that did not provide predefined lists of significant QTL-gene pairs, LD-based clumping was performed using study-specific *p* value thresholds to define candidate instruments and using an LD threshold of *r*^2^ = 0.001 over a 10-Mb window. In cases where selected QTL instruments were not present in the corresponding disease GWAS summary statistics, we searched for suitable LD proxy variants using a European ancestry reference from the 1000 Genomes Project.^40^ Proxy variants were identified using the *LDlinkR* R package (v.1.4.0), which interfaces with the web-based LDlink resource.

To minimize bias due to weak instruments, we assessed whether the instruments were strongly associated with the exposure by leveraging the F statistic for each instrument and selected only those that were larger than 10 for the subsequent causal inference analyses. The F statistic was calculated from summary-level data as beta^2^/SE^2^, where beta is the effect size and SE is the standard error.^59^ If only a single instrument remained after clumping, harmonization, and exclusion of weak instruments based on the F statistic, causal effects were estimated using the Wald ratio method. When at least two independent genetic instruments were available, causal effects were estimated using the IVW method, which performs a random-effects meta-analysis of the Wald ratios for each genetic instrument. Sensitivity analyses included Cochran’s Q test for heterogeneity.

## Results

### Genome-wide genetic overlap and causal association

**Figure 1:**
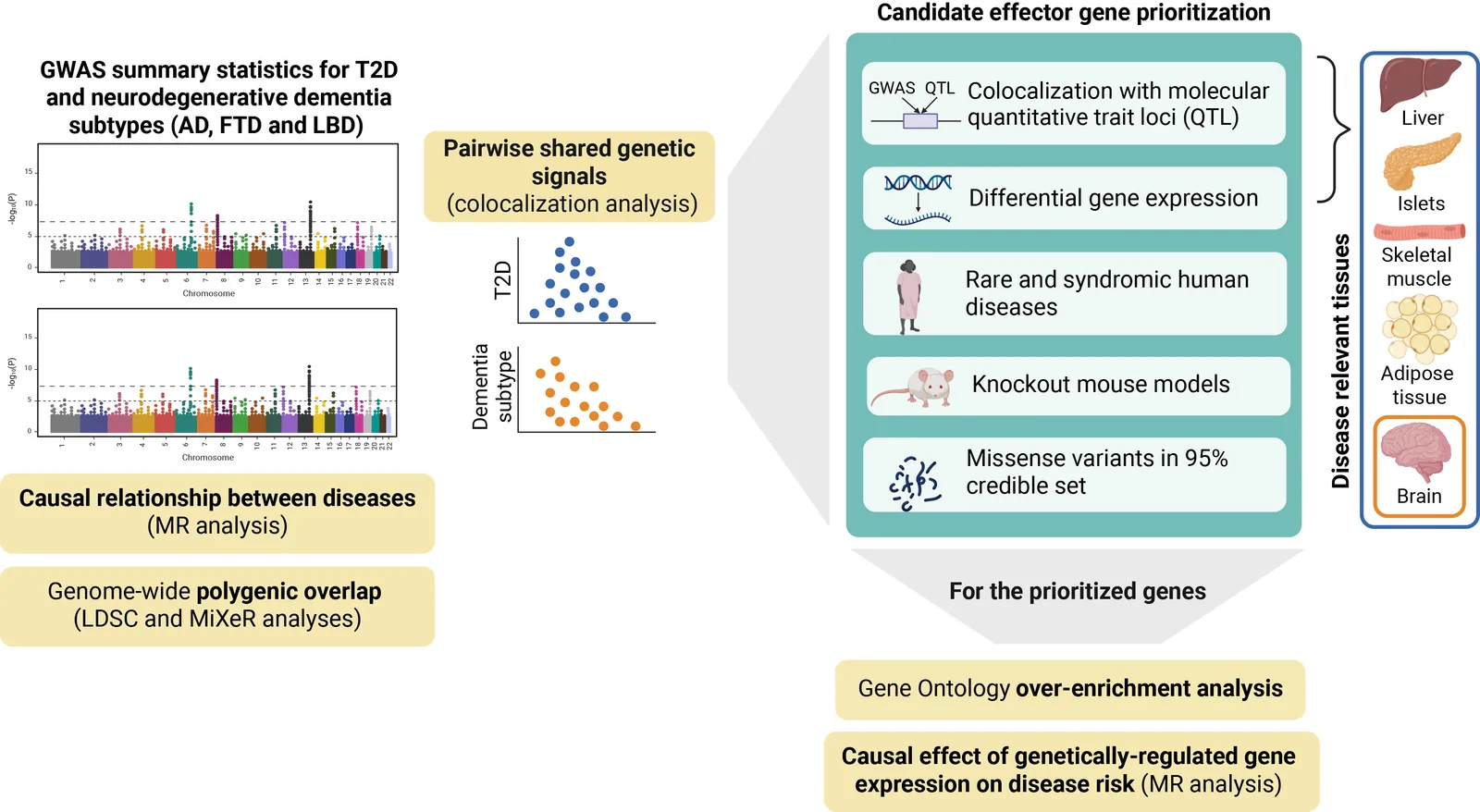
Study design We leveraged publicly available genome-wide association studies (GWASs) for three neurodegenerative dementia subtypes (Alzheimer disease [AD], Lewy body dementia [LBD], and frontotemporal dementia [FTD]) and type 2 diabetes (T2D) to investigate the genetic relationship between these conditions. We first assessed genome-wide genetic relationships between T2D and each dementia subtype separately using linkage disequilibrium score regression (LDSC), MiXeR polygenic overlap analyses, and bidirectional Mendelian randomization (MR). We then performed pairwise statistical colocalization analyses between T2D and each dementia subtype to identify shared association signals. Within the shared genomic regions, we prioritized candidate effector genes by integrating multiple lines of functional genomics evidence from disease-relevant tissues supporting their involvement in each condition. The tissue relevant to dementia (brain) is highlighted with an orange box, while the tissues relevant to T2D are highlighted with a blue box. For the prioritized candidate effector genes, we conducted pathway enrichment analyses and further causal inference using Mendelian randomization to evaluate the effects of genetically regulated gene expression on disease risk. This workflow enabled the identification of both dementia subtype-specific shared loci and loci shared across multiple dementia subtypes. The schematic in this figure was created with BioRender.

We first sought to assess genome-wide genetic overlap between T2D (*N*_cases_ = 242,283; *N*_controls_ = 1,569,734) and three neurodegenerative forms of dementia: AD (*N*_cases_ = 21,982; *N*_controls_ = 41,944),^34^ LBD (*N*_cases_ = 2,591; *N*_controls_ = 4,027),^35^ and sporadic FTD (*N*_cases_ = 3,756; *N*_controls_ = 11,233)^36^^,^^37^ (Figure 1). We restricted our analyses to individuals of European genetic ancestry, due to neurodegenerative dementia GWAS data availability.

We conducted LDSC regression analyses^39^ and found no evidence of genome-wide genetic correlation between T2D and any dementia subtype (Table S2). We next applied MiXeR^41^ to assess whether T2D and dementia subtypes shared polygenic architecture beyond what is captured by genome-wide genetic correlation (Table S3; Figure S1). T2D was estimated to be highly polygenic, with approximately 3,461–3,655 trait-associated variants explaining 90% of SNP heritability. The only evidence of polygenic overlap was observed for T2D-FTD, with an estimated 152 ± 102 shared variants and a Dice coefficient (a measure of overlap between two sets) of 0.076 ± 0.044. MiXeR model comparison supported the best-fitting intermediate-overlap model over both the minimum-overlap model and the maximum-overlap model. However, the MiXeR-estimated genetic correlation was close to zero (*r*_g_ = −0.006 ± 0.022), consistent with the LDSC estimate and suggesting mixed-direction pleiotropy rather than consistently concordant effects. The estimated AD and LBD polygenicity were very low, leading to unstable and non-interpretable estimates.

Genetic liability to AD was previously associated with a lower risk of T2D (odds ratio [OR] = 0.97, 95% confidence interval [CI] = 0.94–0.99, *p* = 4.8 × 10^−3^) with no evidence of directional pleiotropy.^60^ Here, we have expanded this analysis to investigate potential causal relationships between T2D and the other dementia subtypes by performing bidirectional two-sample MR^59^ analyses. We used the IVW method as the primary estimator, complemented by sensitivity analyses (material and methods; Figure S2; Table S4). Apart from the previously reported protective effect of AD genetic liability on T2D risk, we found no additional robust evidence for causal relationships. For FTD, genetic liability showed a nominal association with reduced T2D risk (OR = 0.84, 95% CI = 0.72–0.98, *p* = 0.029). However, there was evidence of heterogeneity across the genetic instruments, and the association did not remain significant after multiple testing correction (FDR = 0.087). We could not test for pleiotropy given the small number of genetic instruments.

### Five genomic regions are shared between neurodegenerative dementia and T2D

To identify shared genetic signals between T2D and the three neurodegenerative forms of dementia considered here, we examined 1-Mb genomic regions centered on genetic association signals (*p* < 5 × 10^−8^) of each trait (Figure 1). By conducting Bayesian colocalization analysis, we assessed the posterior probability of each dementia trait and T2D sharing a causal signal in each of the tested genomic regions (PP4). When using coloc.abf, we found evidence for shared signals (PP4 > 0.8) in three genomic regions (Tables 1 and S5; Figure S1). Because coloc.abf assumes a single causal variant per trait within a tested region, we additionally used PWCoCo to evaluate further shared association evidence while accounting for multiple association signals within a locus and identified two additional shared loci (Table S5).

**Table 1:** Genetic loci shared between type 2 diabetes and neurodegenerative dementia

Dementia form	Lead shared variant	Chromosome	Position	PP4	Effect direction	Top-scoring genes
LBD	rs2230288	1	155206167	0.965	concordant	*GBA*
AD	rs12419690	11	45858584	0.815	opposite	*CRY2*, *PEX16*, *MAPK8IP1*
LBD	rs4787489	16	29995880	0.911	opposite	*INO80E*
FTD	rs9904865	17	44908263	0.998	concordant	*NSF*
AD, FTD	rs429358	19	45411941	1	opposite	*APOE*
LBD	rs769449	19	45410002	0.998		

Overview of the colocalized genomic regions between neurodegenerative dementia and type 2 diabetes (T2D). The genomic positions are in GRCh37. The “effect direction” column shows whether the effect of the colocalized signal has opposite or concordant direction on neurodegenerative dementia and T2D. LBD, Lewy body dementia; AD, Alzheimer disease; FTD, frontotemporal dementia; PP4, posterior probability of a shared causal variant.

A proof of concept for the robustness of this approach is captured by the finding of the well-established *APOE* region (chr19:45407044–45412653, GRCh37) as a shared genetic signal between dementia and T2D.^61^^,^^62^ Only in this region do we find evidence of colocalization between all analyzed neurodegenerative forms of dementia and T2D (PP4: AD = 1, LBD = 0.998, and FTD = 1) with rs429358 and rs769449 as the lead variants of the shared signal (*r*^2^ = 0.77 in European ancestry) (Table 1). The lead missense variant rs429358 corresponds to c.388T>C (GenBank: NM_000041.4) (p.Cys130Arg [GenBank: NP_000032.1]) in *APOE* and is predicted to be benign by PolyPhen2.^63^ Together with rs7412, corresponding to c.526C>T (GenBank: NM_000041.4) (p.Arg176Cys [GenBank: NP_000032.1]), it determines the *APOE* ε2, ε3, and ε4 alleles. Apart from the *APOE* region, the remaining four colocalized genetic regions exhibit shared association signals between only a single neurodegenerative dementia subtype and T2D. We observed no evidence of association signals in these regions across the other investigated dementia subtypes (PP4 and PP3 <0.2).

For AD, we also identify an additional signal on chromosome 11 with opposite directions of association between AD and T2D, indicating directional discordance in shared genetic effects. For FTD, we find an additional colocalized signal on chromosome 17, with the same direction of effect with T2D. Finally, for LBD, we find two additional shared signals on chromosomes 1 and 16. The colocalized variants on chromosome 1 have the same direction of effect for LBD and T2D, whereas the signal on chromosome 16 shows opposite directions of association for each condition. The shared signal rs2230288 on chromosome 1 corresponds to the *GBA* (MIM: 606463) missense variant c.1226A>G (GenBank: NM_000157.4) (p.Asn409Ser [GenBank: NP_000148.2]) and is predicted to be benign by PolyPhen2.^63^ This gene is associated with Gaucher disease, a metabolic lysosomal storage disorder caused by acid β-glucosidase deficiency. Subtype II (MIM: 230900) of this disease leads to decreased glucocerebrosidase activity, progressive neurologic deterioration, and rapidly progressive brainstem degeneration, and subtype III (MIM: 231000) is associated directly with dementia.^64^

### Evidence for 26 genes involved in neurodegenerative dementia and T2D

**Figure 2:**
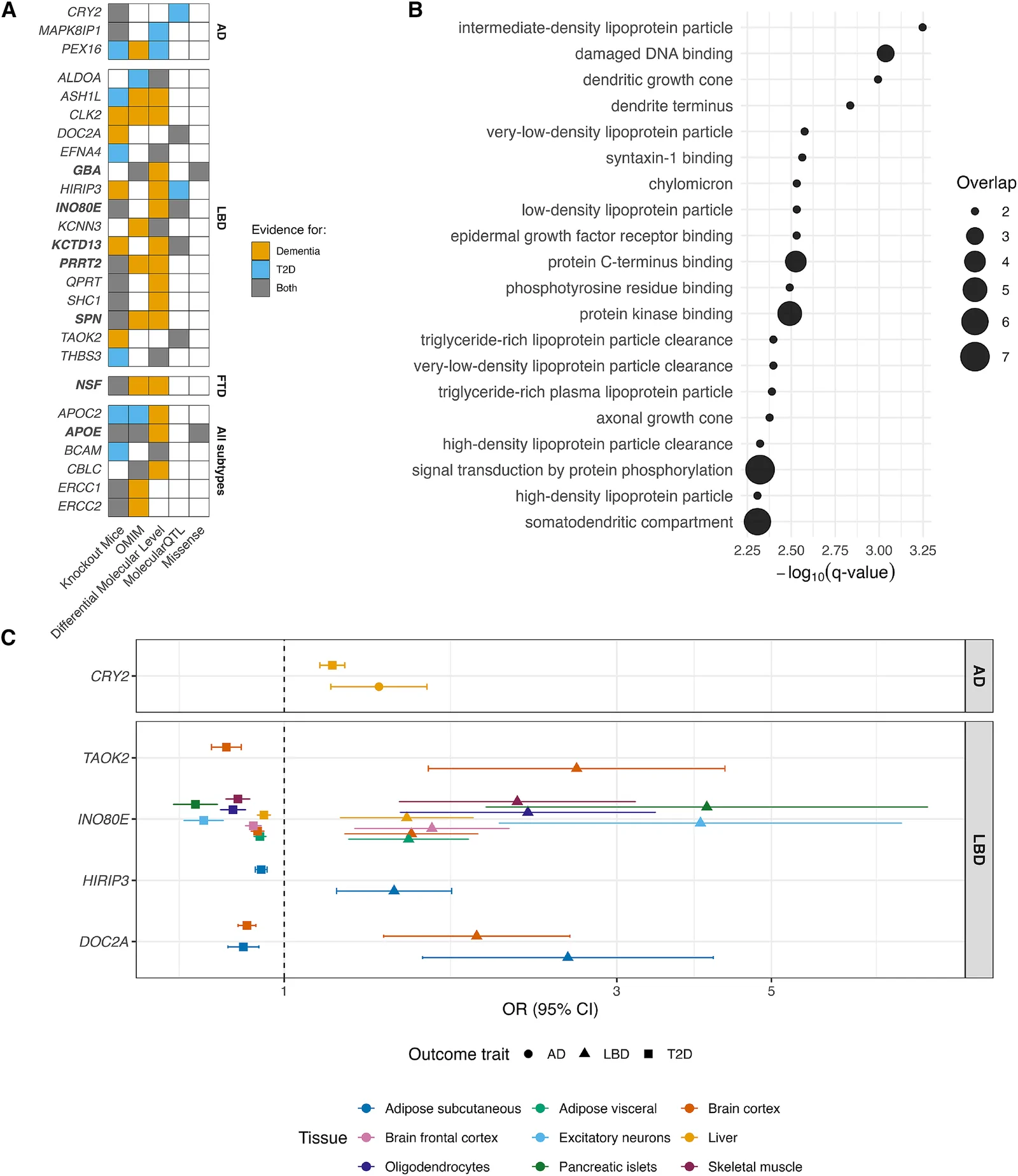
Gene prioritization (A) Prioritized shared effector genes. Overview of the scoring of the 26 genes prioritized by our genomics data-driven approach located in the colocalized regions between T2D and individual neurodegenerative dementia subtypes (AD, Alzheimer disease; LBD, Lewy body dementia; FTD, frontotemporal dementia). Most prioritized genes originate from loci shared between T2D and a single dementia subtype, whereas only genes within the *APOE* locus are shared across all three dementia subtypes. Genes highlighted in bold are the top-scoring genes (score ≥ 4). T2D, type 2 diabetes; QTL, quantitative trait loci. (B) Top 20 Gene Ontology (GO) terms (*q* value <0.05) enriched among the 26 prioritized candidate genes, highlighting biological pathways implicated in the subtype-specific and shared genetic relationships between T2D and neurodegenerative dementias. (C) Results (*q* value <0.05) of the two-sample Mendelian randomization (MR) analysis evaluating the effects of genetically regulated expression of prioritized genes on T2D and the corresponding dementia subtype. The *x* axis shows the odds ratio (OR) per unit increase of gene level and the corresponding 95% confidence interval (CI). This figure was assembled using BioRender.

We sought to prioritize candidate genes shared between neurodegenerative dementia and T2D by scoring all genes located in the identified shared association regions based on five orthogonal lines of evidence (Figure 1; Table S10). In brief, we tested for multi-trait colocalization with molecular QTLs from disease-relevant tissues (Table S6), examined differential gene and protein expression levels in relevant tissues between affected individuals and healthy control subjects (Table S11), identified missense coding variants, and studied phenotypes associated with either condition in rare or syndromic human diseases associated with these genes (Table S8), as well as in knockout mouse models (Table S9) (material and methods). In total, 259 genes were located within the five shared genomic regions (Table S10). We prioritize 26 genes showing at least three lines of evidence pointing to involvement in both neurodegenerative dementia and T2D (Figure 2A; Table S12). Of these, 16 (61.54%) are located in the genomic regions that colocalize between LBD and T2D only (regions on chromosome 1 or 16), one (*NSF* [MIM: 601633]) is located in the genomic region that colocalizes between FTD and T2D only (region on chromosome 17), three (*PEX16* [MIM: 603360], *MAPK8IP1* [MIM: 60464] and *CRY2* [MIM: 603732]) are located in the region that colocalizes between AD and T2D only (region on chromosome 11), and six (*APOE*, *APOC2* [MIM: 608083], *BCAM* [MIM: 612773], *CBLC* [MIM: 608453], *ERCC1* [MIM: 126380], and *ERCC2* [MIM: 126340]) are in the *APOE* region on chromosome 19 that colocalizes between all three analyzed forms of dementia and T2D.

### Genetic evidence for a role of lipid regulatory biology in dementia and T2D comorbidity

To glean further insights into the set of 26 genes prioritized by our genomics data-driven approach, we conducted an enrichment analysis using Gene Ontology terms (Table S13). The lead pathways for which we observe gene enrichment were linked to lipoprotein particles (*APOE* and *APOC2* from the chromosome 19 locus; *q* = 5.66 × 10^−4^), including their clearance, and “damaged DNA binding” (*CRY2*, *ERCC1*, and *ERCC2* from the chromosome 16 locus; *q* = 9.18 × 10^−4^) (Figure 2B). Enriched pathways with a larger number of overlapping genes were associated with broader biological processes, including “positive regulation of cellular metabolic process” (*q* = 0.024, overlap = 11), “organelle organization” (*q* = 0.0056, overlap = 14), and “protein binding” (*q* = 0.025, overlap = 25) (Table S13).

Since the genes prioritized by our genomics data-driven approach were enriched in lipid-related pathways, we investigated whether the genomic regions shared between neurodegenerative dementia and T2D are associated (*p* < 5 × 10^−8^) with circulating lipid traits using GWAS summary data from the GLGC (Table S14).^38^ The chromosome 19 *APOE* locus showed the expected associations with all analyzed lipid traits, in line with the well-established effects of *APOE* variants in systemic lipid metabolism.^65^^,^^66^ In addition, the chromosome 16 locus shared between LBD and T2D was associated with triglyceride and HDL-C levels. Variants in the remaining shared regions were neither significantly associated with circulating lipid traits nor in strong (*r*^2^ > 0.5) LD with lead variants from lipid-associated genomic loci identified in the lipid GWAS meta-analysis.

### Prioritized shared genes have mostly directional discordant causal effects on neurodegenerative dementia and T2D

For the genes prioritized by our genomics data-driven approach that showed evidence of colocalization between dementia, T2D, and an eQTL dataset from a disease-relevant tissue, we performed two-sample MR between genetically regulated gene expression and disease risk (Table S15).^59^ This analysis aimed to uncover directional evidence of involvement of the prioritized effector genes in the comorbidity. We find evidence of a causal effect of genetically regulated expression of five genes on the risk of dementia or T2D (Figure 2C).

We find that genetic variants associated with higher gene expression in disease-relevant tissues are associated with higher risk of LBD but lower risk of T2D, suggesting pleiotropic effects with opposing phenotypic consequences (Table 2). This is in line with the opposite direction of effect of the shared signal on chromosome 16 for LBD and T2D, where *DOC2A* (MIM: 604567), *HIRIP3* (MIM: 603365), *INO80E*, and *TAOK2* (MIM: 613199) are located. For *CRY2*, which originates from a locus shared between T2D and AD, genetic variants associated with increased expression of this gene in the liver have a risk-increasing effect on both AD (OR = 1.37, *q* = 1.42 × 10^−4^) and T2D (OR = 1.17, *q* = 3.89 × 10^−14^). Knockout mouse models for *CRY2* show phenotypes associated with both dementia and T2D, including impaired glucose tolerance, increased insulin sensitivity, decreased total body fat amount, and decreased locomotor activity (Table S9).

**Table 2:** Effect direction of putative shared causal genes

Gene	Region	Tissue	SNP	Effect direction		
				eQTL	T2D	Dementia subtype
*CRY2*	chr11	liver	rs12805422 (G)	0.18	0.029	0.056
*DOC2A*	chr16	subcutaneous adipose	rs4077410 (A)	0.15	−0.021	0.14
*DOC2A*	chr16	brain cortex	rs12921996 (C)	0.24	−0.029	0.15
*HIRIP3*	chr16	adipose tissue	rs8054556 (G)	0.39	−0.029	0.14
*INO80E*	chr16	brain cortex	rs8054556 (G)	0.39	−0.029	0.14
*INO80E*	chr16	pancreatic islets	rs8054556 (G)	0.1	−0.029	0.14
*INO80E*	chr16	oligodendrocytes	rs8054556 (G)	0.17	−0.029	0.14
*INO80E*	chr16	liver	rs4787491 (A)	0.33	−0.022	0.13
*INO80E*	chr16	visceral adipose	rs9932196 (G)	0.37	−0.03	0.15
*INO80E*	chr16	skeletal muscle	rs7193532 (G)	0.19	−0.029	0.14
*INO80E*	chr16	cerebellum	rs9925102 (T)	0.74	−0.03	0.15
*INO80E*	chr16	brain cortex	rs3814880 (T)	0.58	−0.03	0.15
*INO80E*	chr16	frontal cortex	rs3814880 (T)	0.29	−0.03	0.15
*INO80E*	chr16	excitatory neurons	rs12921996 (C)	0.11	−0.029	0.15
*TAOK2*	chr16	brain cortex	rs4788200 (G)	0.15	−0.029	0.14

Overview of the direction of association effect of the genetic variants used as instrumental variables in the Mendelian randomization analysis conducted to estimate the causal effect of genetically regulated increased gene expression of prioritized genes in disease-relevant tissues on dementia and type 2 diabetes (T2D) risk.

For *INO80E*, we identify causal effects of genetically regulated gene expression on both LBD and T2D risk across a wide range of disease-relevant tissues, including multiple brain regions, pancreatic islets, liver, adipose tissue, and skeletal muscle. We observed evidence for both conditions across all of these tissues, including effects of genetically predicted gene expression in metabolic tissues on LBD risk (Figure 2). Across all tissues and cell types examined, the direction of effect was consistent: higher *INO80E* expression had a risk-increasing effect on LBD and a protective effect against T2D (Table 2). These cross-tissue consistent findings show that the opposing effects of genetically predicted *INO80E* expression on LBD and T2D are detectable across both central nervous system and peripheral metabolic tissues rather than being restricted to a single-tissue context for either condition. *INO80E* knockout mice have increased body weight as well as abnormal locomotor activity (Table S9). When querying a differential protein abundance resource in the plasma for dementia,^53^ INO80E was underexpressed in the plasma of dementia individuals in comparison with healthy control subjects (log fold change = −0.05, FDR = 9.59 × 10^−4^) (Table S11), which is opposite to the MR results mentioned above, where genetics variants associated with higher expression of *INO80E* in multiple primary disease-relevant tissues have a risk-increasing effect on LBD. This discrepancy might be rooted in the fact that the differential protein abundance dataset is based on cases of AD and FTD, and not on LBD, or might signal mechanistic differences between gene expression and protein abundance due to post-translational changes.

## Discussion

Within the context of global population aging, understanding how metabolic and neurodegenerative diseases intersect is increasingly critical. Dementia and T2D frequently co-occur in older individuals, yet the extent to which this comorbidity reflects shared genetic susceptibility beyond risk mediated by vascular effects remains unclear. By focusing on dementia forms with predominantly neurodegenerative etiologies, we aimed to investigate the as yet less well-characterized shared genetic mechanisms with T2D, beyond the well-characterized cerebrovascular risk pathways. Enrichment analyses have indeed highlighted differences in the genetic architecture between all-cause and vascular dementias, with the former showing enrichment for amyloid-β and amyloid precursor protein processing pathways, and the latter showing enrichment for metabolic response pathways, including glucose and carbohydrate response.^33^ Hence, we did not include vascular dementia in our analysis, as it would likely capture mediated effects of diabetes on cerebrovascular disease rather than shared genetic susceptibility. Future work could explicitly model vascular dementia as an outcome in mediation-aware frameworks to disentangle direct and indirect effects of T2D on cognitive decline.

We based our analyses on data from large-scale case-control GWASs of individuals diagnosed with dementia instead of population-based proxy case and control groups relying on parental dementia status, as multiple recent studies have highlighted challenges associated with using proxy data in genetic studies.^67^^,^^68^ Restricting the data to clinically diagnosed dementia resulted in smaller sample sizes for the neurodegenerative dementia GWAS compared to the T2D GWAS. GWAS data with clinically diagnosed individuals with different dementia subtypes across global populations are needed examine the transferability of findings beyond European genetic ancestry. Sex differences are well documented in both neurodegenerative dementia and T2D^69^^,^^70^; thus, future work should also incorporate sex-stratified analyses to improve resolution of results and potentially identify sex-specific genetic risk factors underlying this comorbidity. Finally, our study prioritizes candidate effector genes for evaluation in large, well-characterized cohorts with individual-level genetic data to assess their biological and clinical relevance.

Previous studies have reported evidence of shared genetic etiology between AD and T2D, including genome-wide genetic correlation and variant overlap.^29^^,^^71^ Our genome-wide analyses indicate that T2D and neurodegenerative dementias do not share substantial genome-wide genetic architecture, and we found little evidence supporting direct causal relationships between T2D, LBD, and FTD. Instead, the genetic relationship is driven by a limited number of shared loci, most of which are dementia subtype specific. Although MiXeR identified modest polygenic overlap between T2D and FTD, the near-zero genetic correlation indicates mixed-direction genetic effects rather than broadly concordant pleiotropy. This interpretation is further supported by our locus-level colocalization and molecular QTL analyses, which revealed shared loci with predominantly opposing effects on metabolic and neurodegenerative disease risk, and by MR analyses showing largely discordant effects of genetically regulated gene expression across the two condition groups.

We identify specific shared genetic signals and systematically characterize shared candidate genes and pathways in these regions beyond cerebrovascular pathways. Moreover, we provide evidence for shared genetic signals between the less well-characterized comorbidities of LBD and FTD with T2D. We identify *APOE* as one of the top-scoring genes and the only region shared between all studied dementia forms and T2D. The other four identified genetic signals are shared only between one form of neurodegenerative dementia and T2D, suggesting that distinct neurodegenerative processes may intersect with metabolic dysfunction through partially overlapping but non-identical pathways. Our genomics-driven framework reveals lipid regulatory biology as a central node linking the shared genetic signals of neurodegenerative dementia and T2D. This observation aligns with previous preclinical work showing an association of lipids with both dementia and T2D.^72^^,^^73^ As larger GWASs of clinically diagnosed dementia subtypes become available, it will be important to reassess the extent of shared genetic architecture between T2D and neurodegenerative dementia, as the currently available studies may have limited power to detect additional shared genetic components.

While overall T2D genetic risk is associated with increased dementia risk,^32^ the identified shared signal in the *APOE* locus led by the missense variant rs429358, which promotes the high-risk APOE ε4 isoform, shows opposing effect direction on dementia and T2D risk. In the same study, the effect of the T2D genetic risk without rs429358 on increased risk of AD was stronger than when including rs429358, in line with our observation. Two further shared regions, which colocalize only between LBD and T2D, also exhibit opposing directions of effect on neurodegenerative and metabolic risk, a pattern that is recapitulated at the molecular trait levels. These locus-level trade-offs do not contradict the observed epidemiological comorbidity between dementia and T2D, as many genes have tissue-specific effects and genetic effects may manifest at different life stages. For instance, variants promoting dyslipidemia or hyperinsulinemia could confer transient metabolic advantages early in life but accelerate neurodegenerative damage later in life.^72^

Taken together, our findings suggest that the epidemiological comorbidity between T2D and neurodegenerative dementias is more consistent with localized pleiotropic mechanisms and antagonistic genetic trade-offs than with broadly shared polygenic susceptibility or common pathogenic mechanisms acting in the same direction. The observation that several dementia subtype-specific shared loci and prioritized effector genes exert opposing effects on metabolic and neurodegenerative disease risk underscores that comorbidity is driven by a limited number of biologically informative loci rather than a uniformly aligned genome-wide architecture. By identifying T2D and dementia subtype-specific shared loci, this work provides a mechanistic roadmap for understanding multi-morbidity. As longevity increases, cross-disease genetic analyses will be essential for designing interventions that mitigate rather than redistribute age-related disease risk.

## Data and code availability

The code used to perform the analyses and generate the figures in this study is publicly available at https://github.com/nalu357/T2D_dementia_comorbidity and archived in Zenodo at https://doi.org/10.5281/zenodo.21245342.

## Author contributions

A.L.A. and E.Z. conceptualized the project; all authors reviewed the analysis plan; A.L.A. and A.S. performed the analyses; A.L.A. and A.S. wrote the manuscript; and all authors revised the manuscript.

## Declaration of interests

The authors declare no competing interests.

## Web resources

ConsensusPathDB, http://cpdb.molgen.mpg.de/
GenBank, https://www.ncbi.nlm.nih.gov/genbank/
International Mouse Phenotyping Consortium (IMPC), https://www.mousephenotype.org/
Mouse Genome Informatics (MGI), http://www.informatics.jax.org/
Online Mendelian Inheritance in Man (OMIM), https://www.omim.org/
Rat Genome Database (RGD), https://rgd.mcw.edu/

## Supplemental information

Document S1. Figures S1 and S2

Table S1. Genome-wide genetic correlation using LD score regression between type 2 diabetes and neurodegenerative dementia subtypes

Table S2. MiXeR estimates of polygenic overlap between type 2 diabetes and Alzheimer disease, Lewy body dementia, and frontotemporal dementia

Table S3. Bidirectional two-sample Mendelian randomization results between type 2 diabetes and neurodegenerative dementia

Table S4. Overview of the molecular QTL data used in our study with sample size, the disease of interest, and reference AD, Alzheimer disease; FTD, frontotemporal dementia; LBD, Lewy body dementia; T2D, type 2 diabetes.

Table S5. Colocalized signals (PP4 > 0.8) along with all variants in the 95% credible set resulting from the coloc.abf analysis We have considered a signal as colocalized if it was not in the HLA region.

Table S6. Signals that colocalized between type 2 diabetes, a neurodegenerative dementia form, and an eQTL from disease-relevant tissues

Table S7. HyPrColoc prior.c sensitivity analysis for the regions that colocalize between type 2 diabetes, a neurodegenerative dementia subtype, and a molecular QTL trait

Table S8. List of rare and syndromic human diseases associated with genes in the colocalized regions and with phenotypes related to type 2 diabetes and neurodegenerative dementia

Table S9. List of knockout mouse phenotypes associated with type 2 diabetes and neurodegenerative dementia

Table S10. Scoring of genes located in the colocalized genomic regions between type 2 diabetes and neurodegenerative dementia

Table S11. Differential molecular traits lookup for genes located in the colocalized regions between type 2 diabetes and neurodegenerative dementia

Table S12. Overview of the scoring of the 26 prioritized candidate genes (score ≥4) including the HyPrColoc and Mendelian randomization results

Table S13. Results (*q* value <0.05) of over-representation analysis using Gene Ontology terms on the 26 genes prioritized by our genomics data-driven approach

Table S14. Genetic variants from T2D-dementia shared regions significantly associated with lipid traits from the European subset of the Global Lipids Genetics Consortium GWAS meta-analysis LDL-C, low-density lipoprotein cholesterol; HDL-C, high-density lipoprotein cholesterol; TG, triglycerides; TC, total cholesterol; non-HDL-C, non-high-density lipoprotein cholesterol.

Table S15. Results (FDR < 0.05) of causal inference analysis using Mendelian randomization to assess the causal effect of genetic variants associated with expression of genes in disease-relevant tissues on either type 2 diabetes or neurodegenerative dementia

Document S2. Article plus supplemental information
