# Supplementary material for: Elucidating shared genetic signals between type 2 diabetes and three neurodegenerative dementia phenotypes
Source: HGG Adv. 2026 Sep 1;7(4):100667. doi: 10.1016/j.xhgg.2026.100667 (PMC13594819; doi:10.1016/j.xhgg.2026.100667)
Supplement: Supplementary file 1 [file mmc1.pdf]

HGGA, Volume *7*

Supplemental information

Elucidating shared genetic signals between type 2 diabetes and three neurodegenerative dementia phenotypes

Ana Luiza Arruda, Archit Singh, Ozvan Bocher, Benjamin F. Voight, Andrew P. Morris, and Eleftheria Zeggini

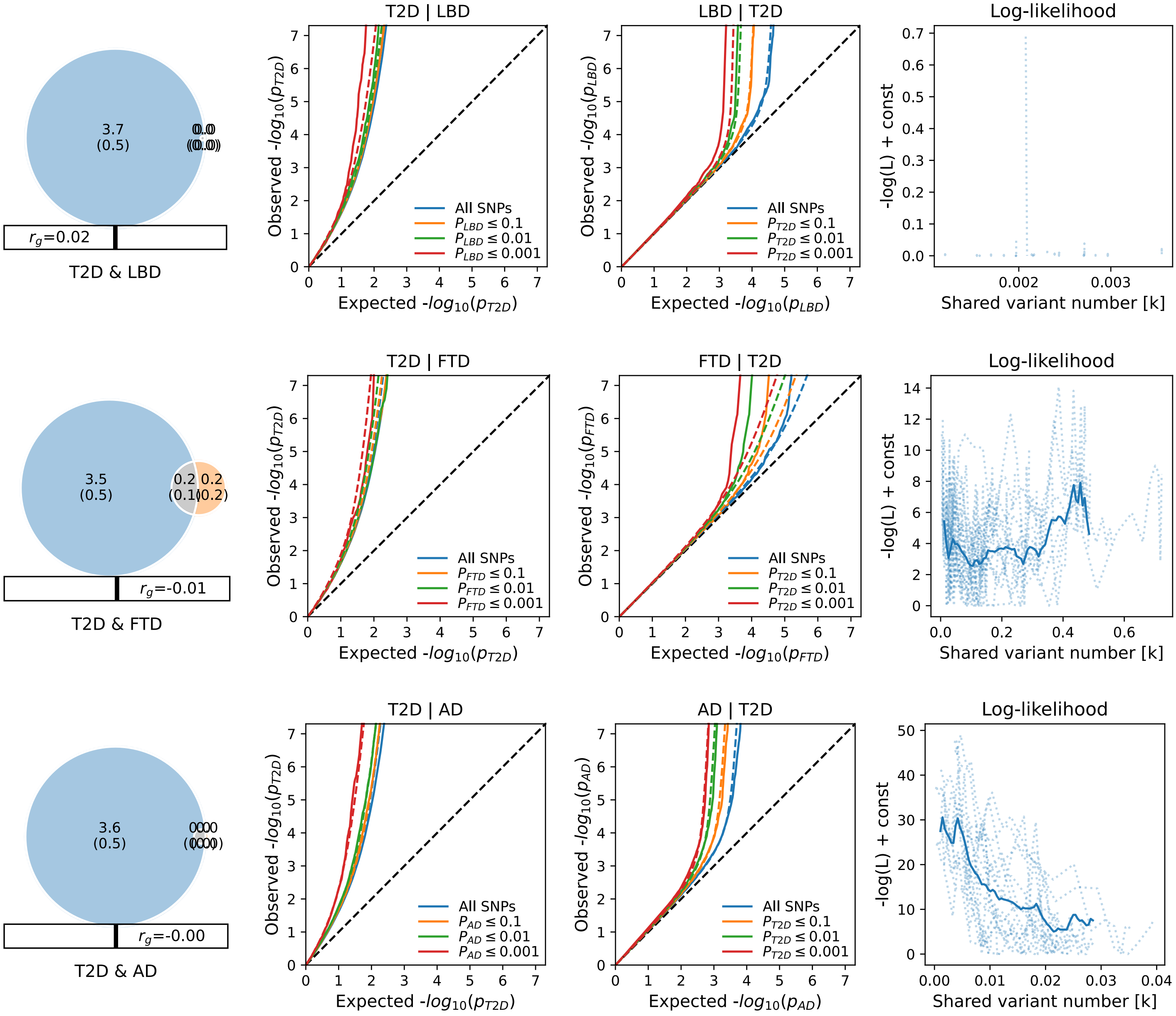

Figure S1: MiXeR analyses of the polygenic overlap between type 2 diabetes (T2D) and three neurodegenerative dementia subtypes: Alzheimer's disease (AD), Lewy body dementia (LBD), and frontotemporal dementia (FTD). Univariate MiXeR analyses (left) estimate the polygenicity and discoverability of each trait. Bivariate MiXeR analyses (middle) compare the observed and expected distributions of association statistics under alternative models of polygenic overlap (minimum, intermediate, and maximum overlap). The Venn diagram summarizes the estimated numbers of trait-specific and shared causal variants explaining 90% of SNP heritability. The right panel shows the profile likelihood across the range of shared variant numbers, with the maximum likelihood estimate corresponding to the peak of the curve. Results were obtained using 20 independent random subsets of approximately two million LD-pruned variants. The best-fitting model was selected using Akaike and Bayesian information criteria (AIC and BIC).

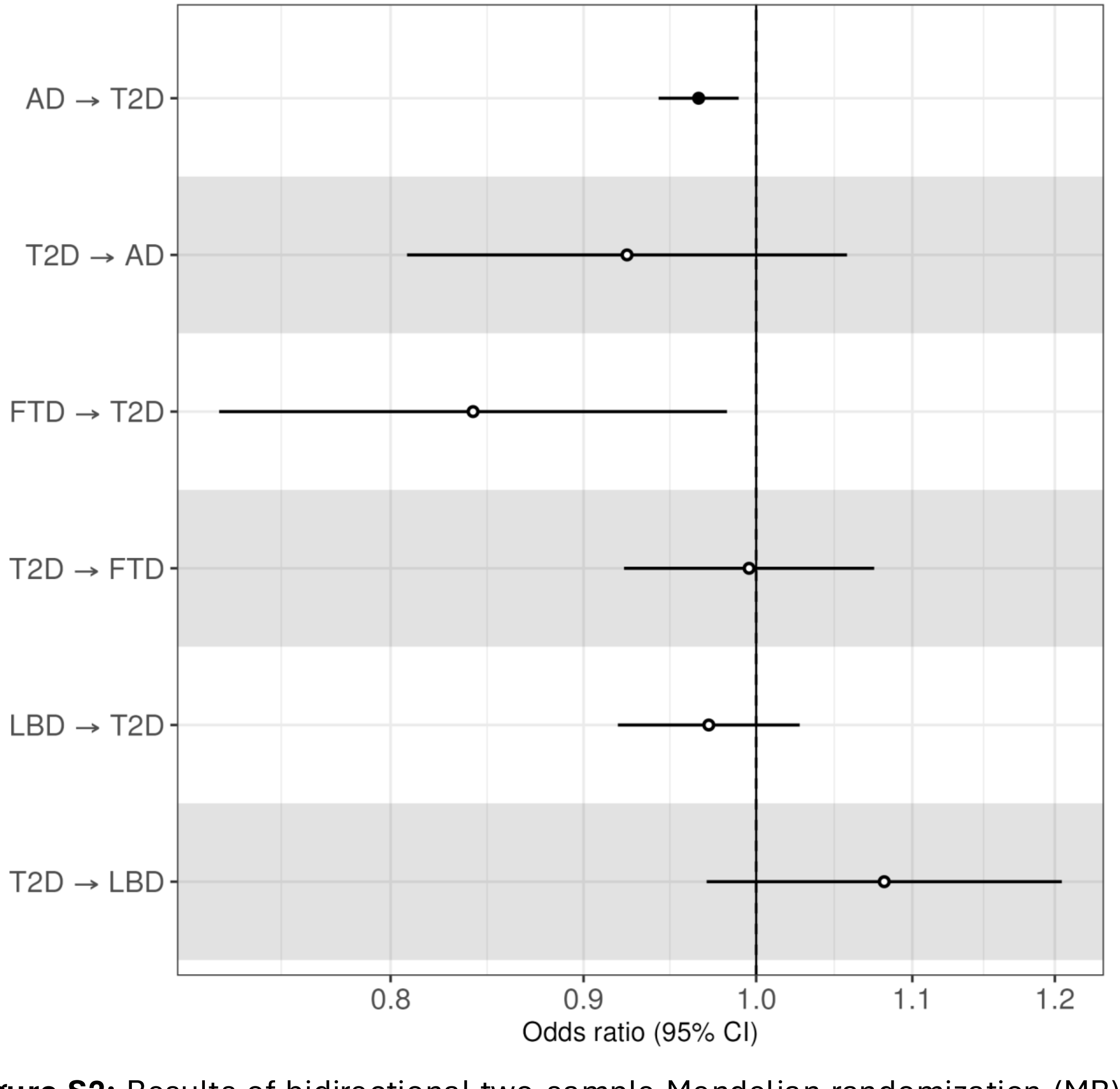

Figure S2: Results of bidirectional two-sample Mendelian randomization (MR) analyses between type 2 diabetes (T2D) and three neurodegenerative dementia subtypes: Alzheimer's disease (AD), Lewy body dementia (LBD), and frontotemporal dementia (FTD). Points represent odds ratios estimated using the inverse variance weighted (IVW) method, and error bars indicate 95% confidence intervals (CI). Results from the sensitivity analyses (MR-Egger, weighted median, MR-PRESSO, and Steiger filtering) are provided in Table S4. Filled symbols indicate associations that remained statistically significant after multiple-testing correction (FDR<0.05).

Figure S2: Regional plots for the genetic loci that colocalize (PP4>0.8) between type 2 diabetes (T2D) and a form of neurodegenerative dementia (AD = Alzheimer's disease, LBD = Lewy body dementia, FTD = frontotemporal dementia).

A) Colocalization region in chromosome 1 between T2D and LBD (PP4=0.965)

B) Colocalization region in chromosome 11 between T2D and AD (PP4=0.815)

C) Colocalization region in chromosome 16 between T2D and LBD (PP4=0.911)

D) Colocalization region in chromosome 17 between T2D and FTD (PP4=0.998)

E) Colocalization region in chromosome 19 between T2D and AD (PP4=1)

F) Colocalization region in chromosome 19 between T2D and FTD (PP4=1)

G) Colocalization region in chromosome 19 between T2D and LBD (PP4=0.998)

A)

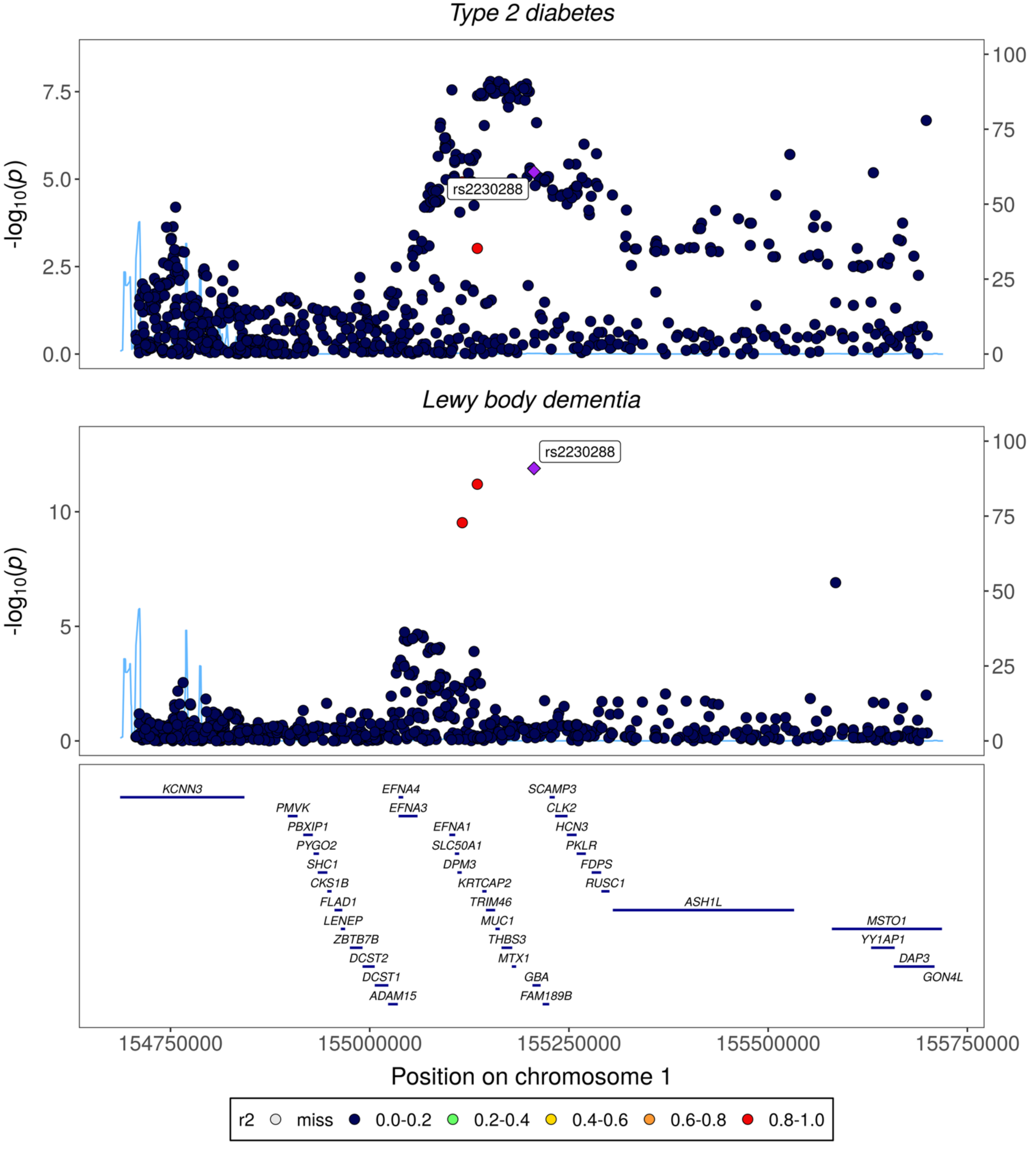

Type 2 diabetes
-log10(p)
Recomb. rate
rs2230288
Lewy body dementia
rs2230288
KCNN3
PMVK
PBXIP1
PYGO2
SHC1
CKS1B
FLAD1
LENEP
ZBTB7B
DCST2
DCST1
ADAM15
EFNA4
EFNA3
EFNA1
SLC50A1
DPM3
KRTCAP2
TRIM46
MUC1
THBS3
MTX1
GBA
FAM189B
SCAMP3
CLK2
HCN3
PKLR
FDPS
RUSC1
ASH1L
MSTO1
YY1AP1
DAP3
GON4L
154750000
155000000
155250000
155500000
155750000
Position on chromosome 1
r2
miss
0.0-0.2
0.2-0.4
0.4-0.6
0.6-0.8
0.8-1.0

B)

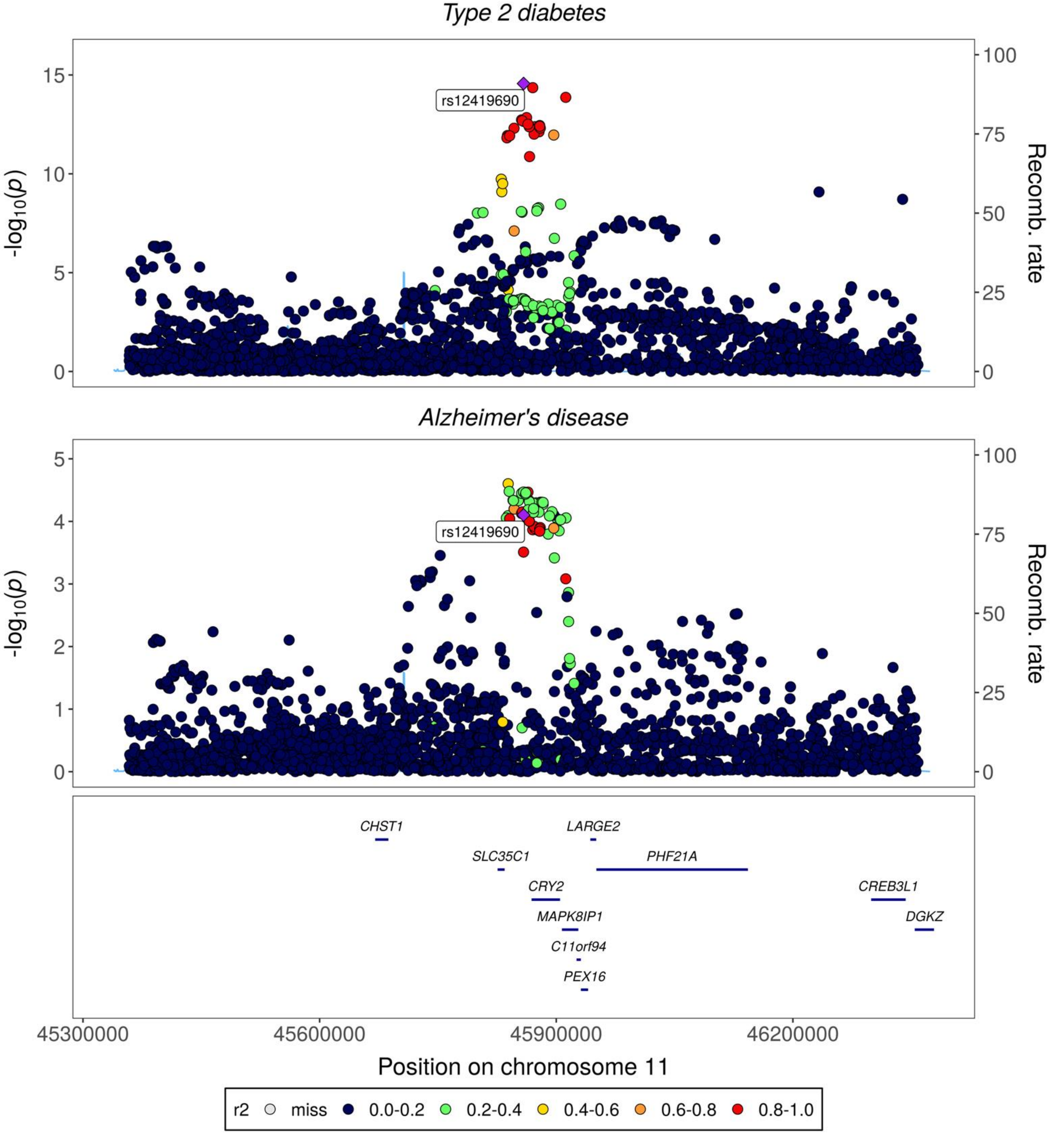

Type 2 diabetes
rs12419690
-log10(p)
Recomb. rate
Alzheimer's disease
rs12419690
-log10(p)
Recomb. rate
CHST1
LARGE2
SLC35C1
PHF21A
CRY2
CREB3L1
MAPK8IP1
DGKZ
C11orf94
PEX16
45300000
45600000
45900000
46200000
Position on chromosome 11
r2
miss
0.0-0.2
0.2-0.4
0.4-0.6
0.6-0.8
0.8-1.0

C)

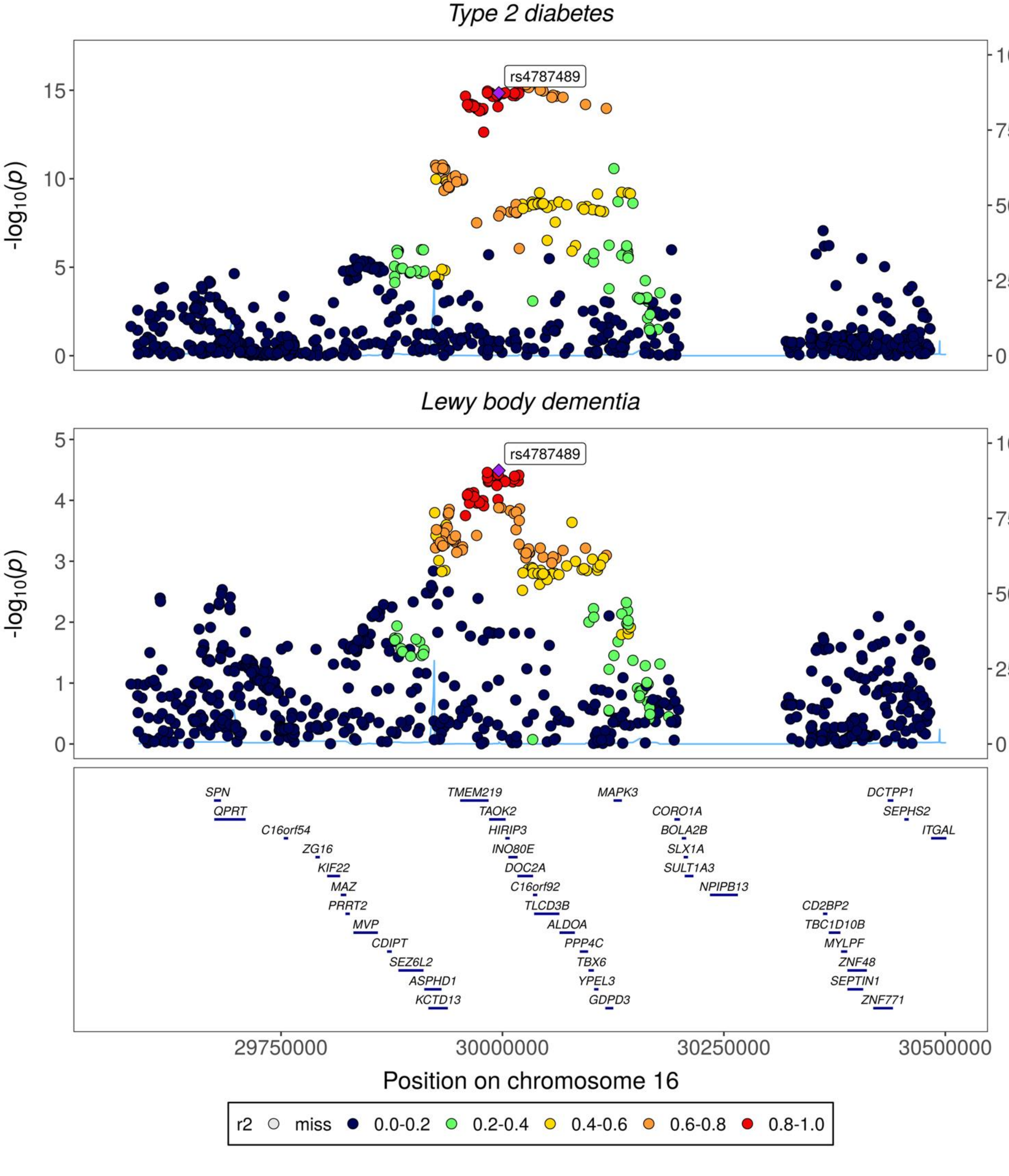
Type 2 diabetes
rs4787489
-log10(p)
Recomb. rate
Lewy body dementia
rs4787489
SPN
QPRT
C16orf54
ZG16
KIF22
MAZ
PRRT2
MVP
CDIPT
SEZ6L2
ASPHD1
KCTD13
TMEM219
TAOK2
HIRIP3
INO80E
DOC2A
C16orf92
TLCD3B
ALDOA
PPP4C
TBX6
YPEL3
GDPD3
MAPK3
CORO1A
BOLA2B
SLX1A
SULT1A3
NPIPB13
CD2BP2
TBC1D10B
MYLPF
ZNF48
SEPTIN1
ZNF771
DCTPP1
SEPHS2
ITGAL
29750000
30000000
30250000
30500000
Position on chromosome 16
r2 miss 0.0-0.2 0.2-0.4 0.4-0.6 0.6-0.8 0.8-1.0

D)

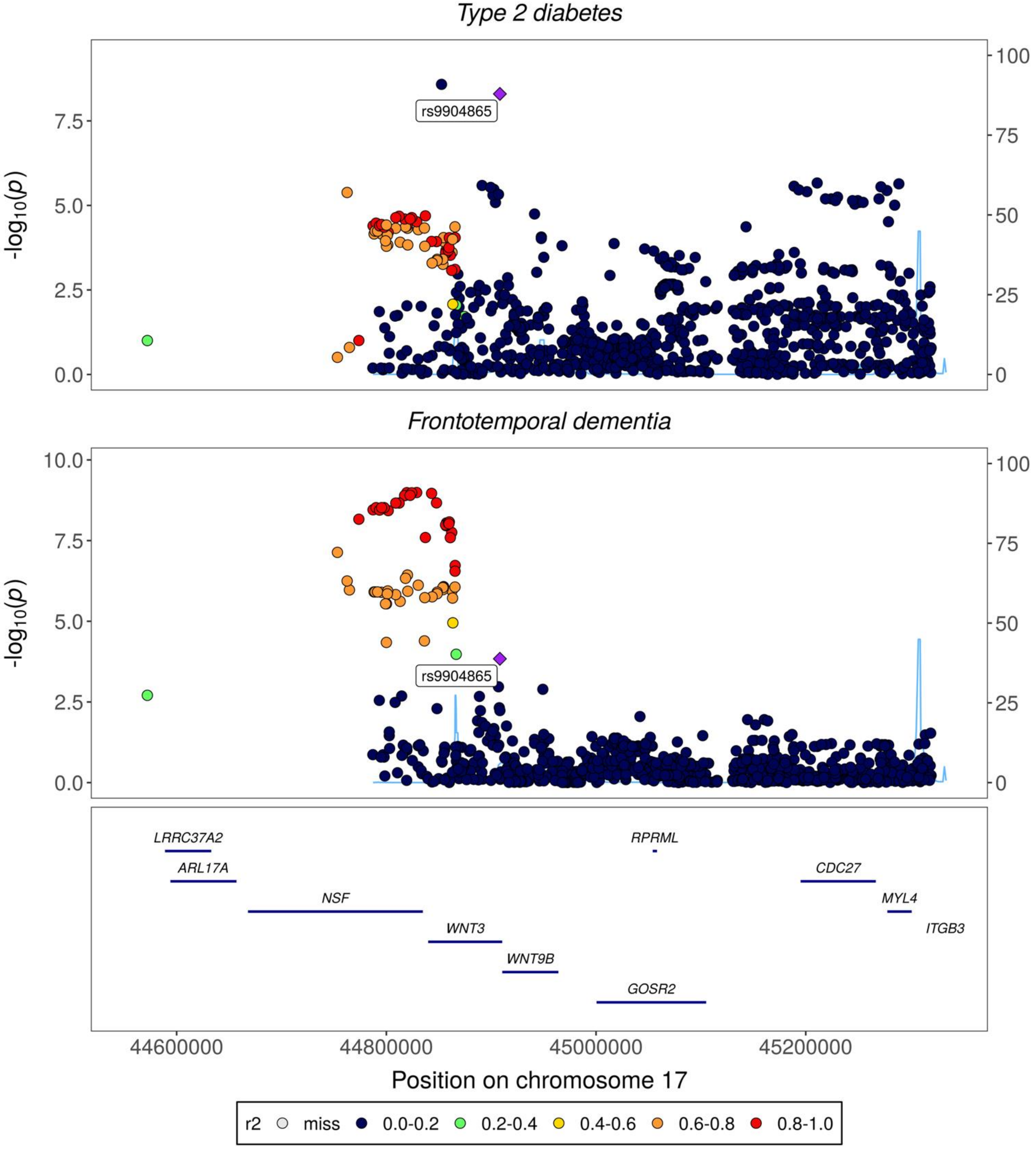

Type 2 diabetes
rs9904865
-log10(p)
Recomb. rate
Frontotemporal dementia
rs9904865
LRRC37A2
ARL17A
NSF
WNT3
WNT9B
RPRML
GOSR2
CDC27
MYL4
ITGB3
44600000
44800000
45000000
45200000
Position on chromosome 17
r2
miss
0.0-0.2
0.2-0.4
0.4-0.6
0.6-0.8
0.8-1.0

E)

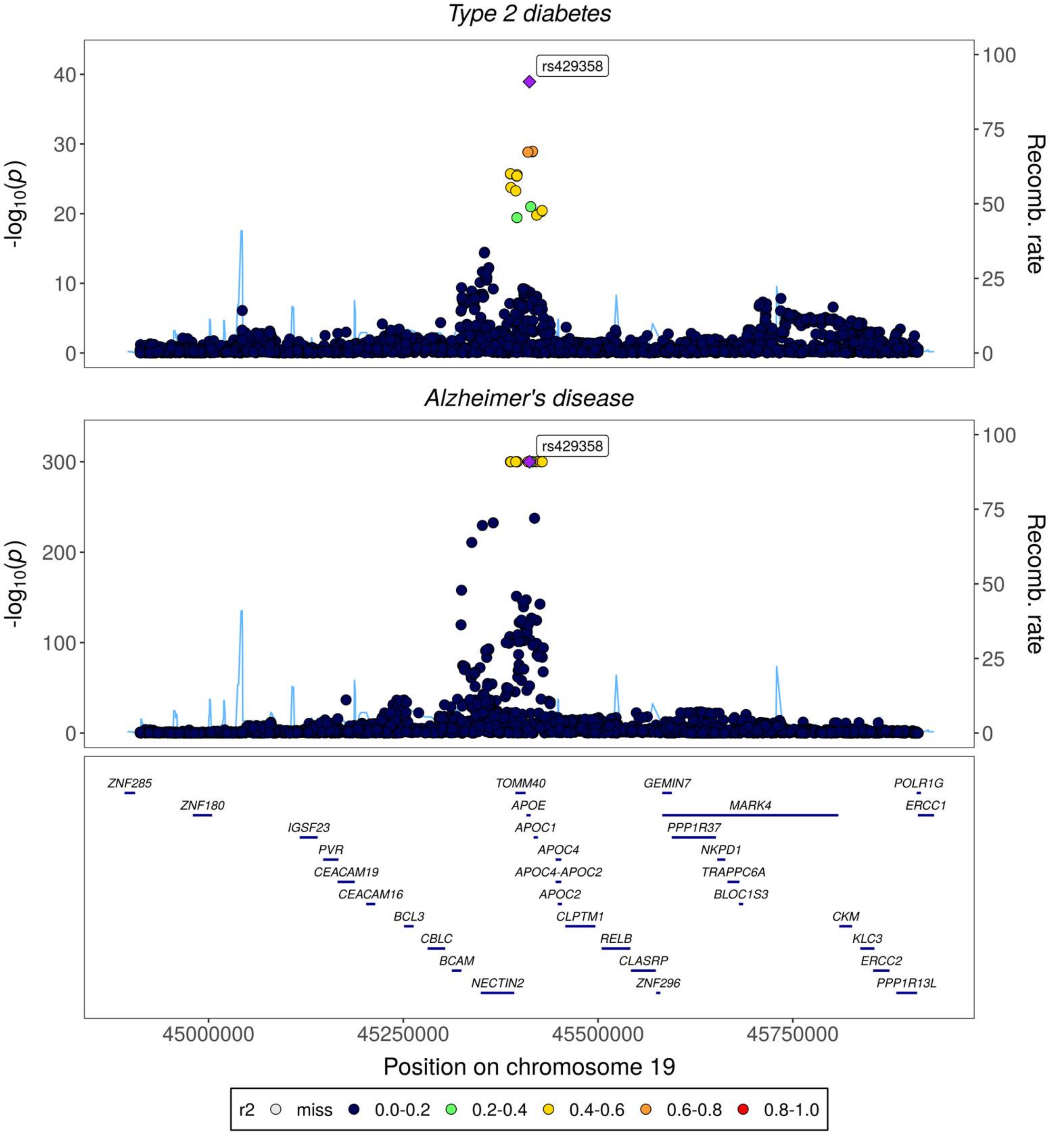

Type 2 diabetes
rs429358
-log10(p)
Recomb. rate
0
10
20
30
40
0
25
50
75
100
Alzheimer's disease
rs429358
-log10(p)
Recomb. rate
0
100
200
300
0
25
50
75
100
ZNF285
ZNF180
IGSF23
PVR
CEACAM19
CEACAM16
BCL3
CBLC
BCAM
NECTIN2
TOMM40
APOE
APOC1
APOC4
APOC4-APOC2
APOC2
CLPTM1
RELB
CLASRP
ZNF296
GEMIN7
MARK4
PPP1R37
NKPD1
TRAPPC6A
BLOC1S3
CKM
KLC3
ERCC2
PPP1R13L
POLR1G
ERCC1
45000000
45250000
45500000
45750000
Position on chromosome 19
r2
miss
0.0-0.2
0.2-0.4
0.4-0.6
0.6-0.8
0.8-1.0

F)

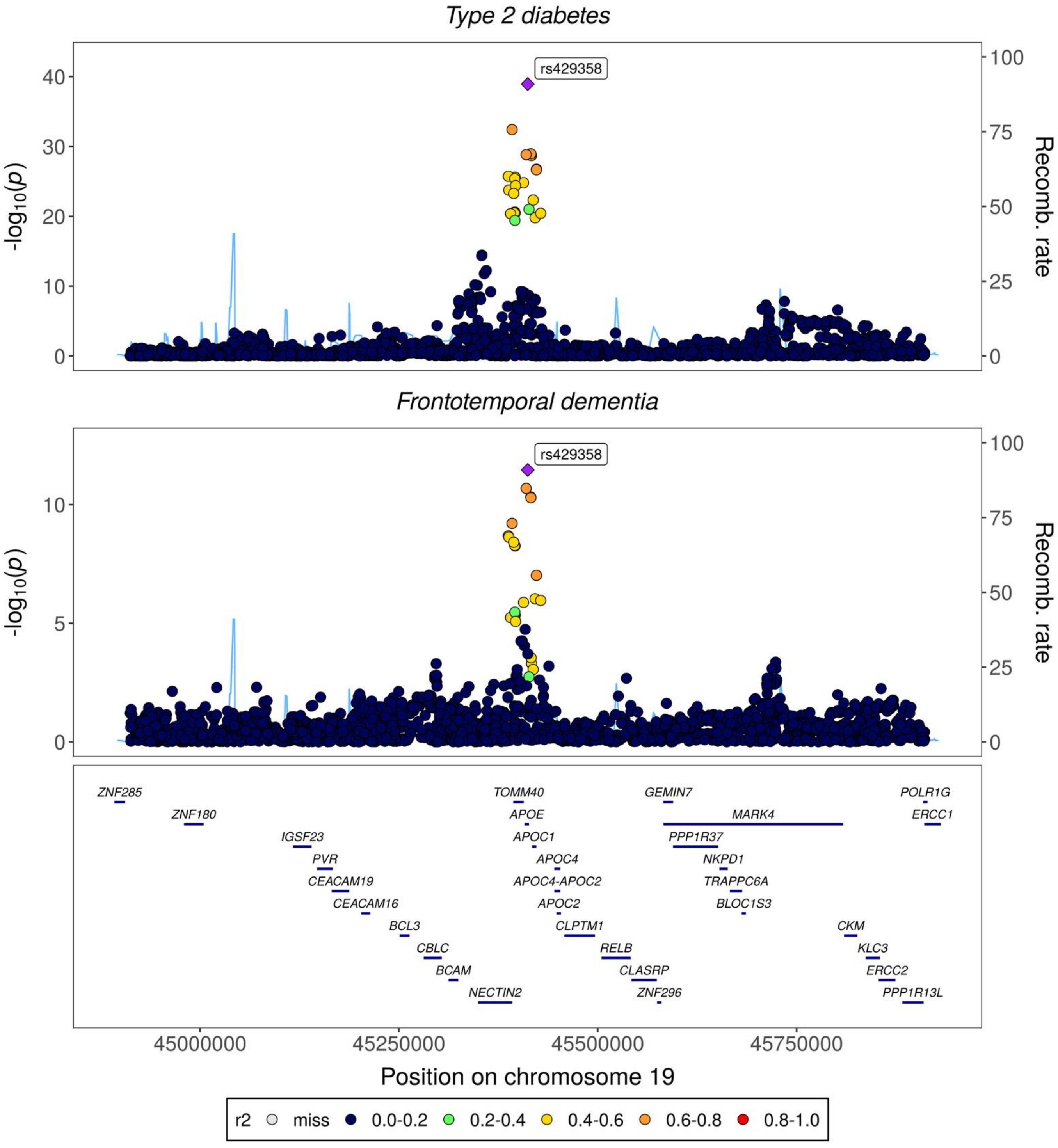
Type 2 diabetes
rs429358
-log10(p)
Recomb. rate
Frontotemporal dementia
rs429358
-log10(p)
Recomb. rate
ZNF285
ZNF180
IGSF23
PVR
CEACAM19
CEACAM16
BCL3
CBLC
BCAM
NECTIN2
TOMM40
APOE
APOC1
APOC4
APOC4-APOC2
APOC2
CLPTM1
RELB
CLASRP
ZNF296
GEMIN7
MARK4
PPP1R37
NKPD1
TRAPPC6A
BLOC1S3
CKM
KLC3
ERCC2
PPP1R13L
POLR1G
ERCC1
45000000
45250000
45500000
45750000
Position on chromosome 19
r2 miss 0.0-0.2 0.2-0.4 0.4-0.6 0.6-0.8 0.8-1.0

G)

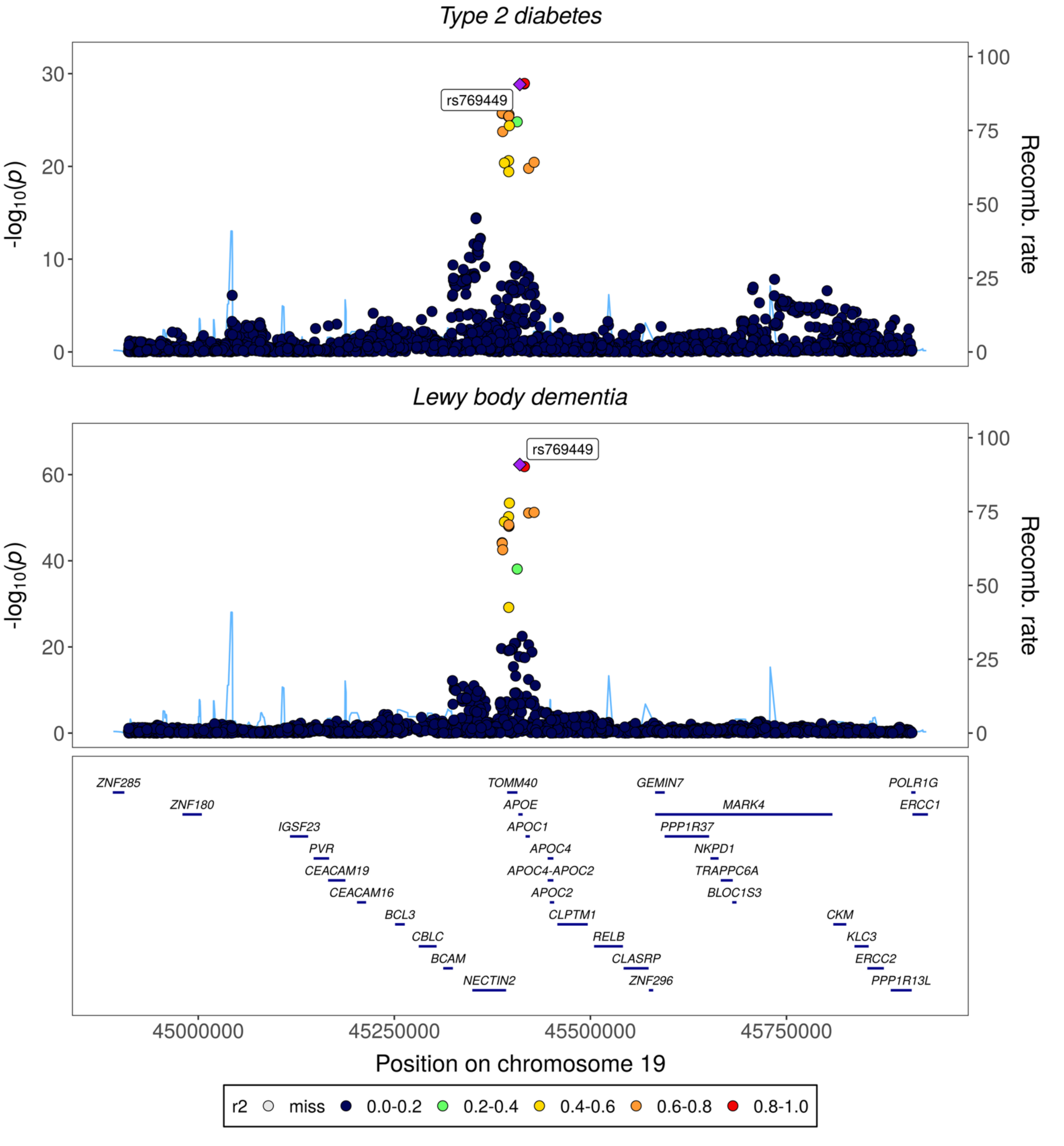

Type 2 diabetes
rs769449
-log10(p)
Recomb. rate
Lewy body dementia
rs769449
ZNF285
ZNF180
IGSF23
PVR
CEACAM19
CEACAM16
BCL3
CBLC
BCAM
NECTIN2
TOMM40
APOE
APOC1
APOC4
APOC4-APOC2
APOC2
CLPTM1
RELB
CLASRP
ZNF296
GEMIN7
MARK4
PPP1R37
NKPD1
TRAPPC6A
BLOC1S3
CKM
KLC3
ERCC2
PPP1R13L
POLR1G
ERCC1
45000000
45250000
45500000
45750000
Position on chromosome 19
r2 miss 0.0-0.2 0.2-0.4 0.4-0.6 0.6-0.8 0.8-1.0
